# Respiratory viral detection in the plasma and cerebrospinal fluid (CSF) of young febrile infants

**DOI:** 10.1111/irv.13250

**Published:** 2024-02-01

**Authors:** Erin G. Nicholson, Vasanthi Avadhanula, Leila C. Sahni, Laura Ferlic‐Stark, Lauren Maurer, Julie A. Boom, Pedro A. Piedra

**Affiliations:** ^1^ Department of Molecular Virology and Microbiology Baylor College of Medicine Houston Texas USA; ^2^ Department of Pediatrics Baylor College of Medicine Houston Texas USA; ^3^ Texas Children's Hospital Houston Texas USA

**Keywords:** enterovirus, febrile infants, parechovirus, respiratory viral infections, serious bacterial infections

## Abstract

**Background:**

Respiratory viral infections are common in febrile infants ≤90 days. However, the detection of viruses other than enterovirus in the blood and cerebrospinal fluid (CSF) of young infants is not well defined. We sought to quantify the occurrence of respiratory viruses in the blood and CSF of febrile infants ≤90 days.

**Methods:**

We conducted a nested cohort study examining plasma and CSF samples from febrile infants 15–90 days via rtPCR. The samples were tested for respiratory viruses (respiratory syncytial virus, influenza, enterovirus, parechovirus, adenovirus, bocavirus). Clinical and laboratory data were also collected to determine the presence of serious bacterial infections (SBI).

**Results:**

Twenty‐four percent (30 of 126) of infants had plasma/CSF specimens positive for a respiratory virus. Enterovirus and parechovirus were the most commonly detected respiratory viruses. Viral positivity was highest in plasma samples at 25% (27 of 107) compared with CSF samples at 15% (nine of 62). SBIs (specifically urinary tract infections) were less common in infants with a sample positive for a respiratory virus compared to those without a virus detected (3% vs. 26%, *p* = 0.008).

**Conclusions:**

Our findings support the use of molecular diagnostics to include the identification of parechovirus in addition to enterovirus in febrile infants ≤90 days. Additionally, these data support the utilization of blood specimens to diagnose enterovirus and parechovirus infections in febrile infants ≤90 days.

## 1 INTRODUCTION

Respiratory viral infections are commonly detected in febrile infants ≤90 days who present to an emergency department (ED) for evaluation.^1^, ^2^, ^3^, ^4^, ^5^ These infants may present with nasal congestion, respiratory distress, and even respiratory failure, but they also present with fever without a source, irritability, and other non‐respiratory symptoms, such as diarrhea, sepsis, and meningitis.^5^, ^6^, ^7^, ^8^ This broad range of presentations substantially overlaps with the clinical signs and symptoms of infants who present with serious bacterial infections (SBIs) and creates a diagnostic dilemma for clinicians.^2^, ^5^, ^9^, ^10^ In an effort to discriminate between viral and bacterial infections in febrile infants ≤90 days, the usage of molecular diagnostics for viral identification are increasing.^2^, ^11^, ^12^

Recently, prospective studies have shown that more than half (58%–70%) of febrile infants ≤90 days will have a virus detected from their nasal secretions via molecular diagnostic testing upon presentation to ED.^5^, ^8^ Viral testing of either respiratory secretions or cerebrospinal fluid (CSF) is often used as part of an algorithm to determine if a febrile infant is at high or low risk for SBI.^2^, ^11^, ^12^, ^13^, ^14^ However, to date, viral testing (with the exception of herpes simplex viruses 1 and 2) is rarely performed using an infant's blood. In the 2021 guidelines from the American Academy of Pediatrics (AAP) for the evaluation and management of febrile infants 8–60 days of life, the guidelines state that “it remains unclear how a positive viral test result should influence further laboratory evaluation and management.”^15^ However, they do state that use of molecular diagnostics for testing the CSF for viruses such as enterovirus and parechovirus could improve clinical decision making.^15^ Although CSF viral testing can help differentiate bacterial infections from viral infections in the setting of CSF pleocytosis, obtaining CSF in young infants is invasive, sometimes technically challenging and often disliked by parents.^16^

Prior research has shown that enterovirus is more commonly detected in the blood than in the CSF of young infants; however, there are less data on other respiratory viruses in this population.^17^ To help address this gap, we designed a study to quantify respiratory viral positivity in the plasma and CSF of young infants. Additionally, as a secondary analysis we examined the incidence of SBI, dichotomized by the presence or absence of a respiratory virus in the plasma and/or CSF of febrile infants ≤90 days.

## 2 MATERIALS AND METHODS

### 2.1 Study design

This nested cohort study utilized residual plasma and CSF samples that were collected from April 2014 to October 2014. These samples were collected as leftover specimens after clinical testing was performed as part of a study to determine the incidence of arboviruses in pediatric patients who presented to the Texas Children's Hospital ED in Houston, Texas.^18^ In brief, individuals ages 15 days to 20 years were eligible for enrollment if they presented to the ED with a temperature ≥38°C or had a history of fever (subjective or measured) and underwent plasma and/or CSF collection during their hospital visit. Individuals were excluded if they had been enrolled within the last 7 days for the same illness or if they or their parent/guardian was not able to consent in Spanish or English. For our study, we identified the subset of enrolled infants aged 15 through 90 days of life, for which there were either plasma and/or CSF samples available and a parent/guardian had provided consent for future use. These samples were evaluated for respiratory viruses by molecular diagnostic testing, and relevant clinical data were abstracted through review of the electronic medical record. The study was approved by the Institutional Review Board at Baylor College of Medicine.

### 2.2 Viral testing

Respiratory viral testing of plasma and CSF samples was performed via singleplex real‐time reverse transcriptase polymerase chain reaction (rtPCR). RNA or DNA was extracted from samples using the QIAamp Viral RNA Mini Kit (QIAGEN Sciences, Maryland, USA) and the automated platform QIAcube (QIAGEN, Hilden, Germany). Primers and probes targeted the most common respiratory viruses in febrile infants ≤90 days (rhinovirus and RSV) as well as those thought most likely to disseminate beyond the respiratory mucosa (enterovirus, parechovirus, adenovirus, and bocavirus).^5^, ^19^, ^20^, ^21^ These specific pathogens were chosen because they are the most common pathogens detected in the respiratory tract of febrile infants ≤90 days (rhinovirus and RSV) and they are the most likely respiratory pathogens to cause symptoms beyond the respiratory tract in immunocompetent children (enterovirus, parechovirus, adenovirus, and bocavirus).^5^ We limited the number of viral tests in this study due to limited volumes of samples available. All samples were tested in a CLIA‐certified respiratory virus molecular diagnostic laboratory. The methods used to differentiate enterovirus and rhinovirus are based on cycle threshold (CT) values and have been previously described.^5^

### 2.3 Clinical data collection

A chart review was performed via the electronic medical record for each infant included in our cohort. Demographics, physical exam findings, culture data, other laboratory results, and treatment were recorded in a standardized electronic form housed in REDCap™, an electronic data capture software program hosted at Baylor College of Medicine in Houston, Texas.^22^, ^23^

### 2.4 SBI criteria

For this study, we defined SBI as bacterial UTI (bUTI), bacteremia, and bacterial meningitis. Infants with SBIs were identified via chart review and the definitions for each SBI are as follows: (1) bUTIs were defined as having >50,000 colony‐forming units (CFU)/mL of bacterial growth in a urine sample obtained via catheterization or having >10,000 CFU/mL of bacterial growth in the same sample type with either pyuria or bacteriuria on microscopy^24^, ^25^; (2) bacteremia and bacterial meningitis were defined by the growth of a pathogen from their respective samples. Contaminants such as coagulase‐negative *Staphylococcus* and *Corynebacterium* species were not considered pathogens unless repeat positive cultures were obtained. Additionally, infants who returned to our institution within 2 weeks of discharge with evidence of a bacterial infection that met the above criteria were counted as having an SBI. These infections were included to capture the potential for a missed SBI during the initial evaluation; however, we recognize that new infections could arise during this time period as well.

### 2.5 Statistical analysis

The primary objective was to describe the frequency of respiratory virus positivity in the plasma and CSF of febrile infants 15–90 days old. The secondary objective was to compare the proportion of SBIs between those positive for a respiratory virus and those negative for a respiratory virus. Demographic and clinical data were also compared across the three groups: those with only plasma samples available, those with only CSF samples available and those with both sample types available. These groupings were compared as proportions via contingency table analysis (chi‐square test or Fisher's exact test) or as means/medians via one‐way ANOVA or Kruskal–Wallis rank test as appropriate. Differences were considered statistically significant at a *p*‐value of <0.05. Statistical analysis was performed using R (R Foundation for Statistical Computing, version 4.2.2) and Stata™ (StataCorp, Version 14.2) statistical software.^26^, ^27^

## 3 RESULTS

### 3.1 Demographics and sample type

A total of 126 infants 15–90 days of life with 107 plasma and 62 CSF samples available were included in this assessment. Forty‐three infants (34%) had both plasma and CSF samples, 64 infants (51%) had only plasma samples, and 19 infants (15%) had only CSF samples. The median age of all enrolled infants was 42 days and 51% were male (Table 1). The median age was significantly higher in infants with only plasma samples available (*p* = <0.001). Consistent with published diagnostic algorithms, only 41% (39 of 95) of infants >29 days of life had CSF obtained.^10^

**TABLE 1:** Demographics.

		Only plasma samples available *n* = 64	Only CSF samples available *n* = 19	Plasma and CSF samples available *n* = 43	All infants *n* = 126	*p*‐value
Age, median days [range]		54 [15–89]	34 [17–74]	33 [16–80]	42 [15–89]	<0.001
Male, *n* (%)		35 (55%)	7 (37%)	22 (51%)	64 (51%)	0.4
Race, *n* (%)	White	44 (69%)	17 (89%)	37 (86%)	98 (78%)	0.3
	Black, African‐American	9 (14%)	2 (11%)	4 (9%)	15 (12%)
	American Indian/Alaskan Native	0 (0%)	0 (0%)	0 (0%)	0 (0%)
	Asian	1 (2%)	0 (0%)	1 (2%)	2 (2%)
	Native Hawaiian/Pacific Islander	0 (0%)	0 (0%)	0 (0%)	0 (0%)
	Multiracial or other	8 (13%)	0 (0%)	1 (2%)	9 (7%)
	Unknown/refused/no response	2 (3%)	0 (0%)	0 (0%)	2 (2%)
Ethnicity, *n* (%)	Hispanic	32 (50%)	10 (53%)	19 (44%)	61 (48%)	0.8
	Non‐Hispanic	32 (50%)	9 (47%)	24 (56%)	65 (52%)	
Recruitment, *n* (%)	Spring: April–May	26 (41%)	5 (26%)	8 (19%)	39 (31%)	0.09
	Summer: June–August	23 (36%)	8 (42%)	16 (37%)	47 (37%)
	Fall: September–October	15 (23%)	6 (32%)	19 (44%)	40 (32%)
Transferred from OSH, *n* (%)		3 (5%)	3 (16%)	5 (12%)	11 (9%)	0.2

Abbreviations: CSF, cerebrospinal fluid, OSH, outside hospital.

### 3.2 Respiratory viral test results

**FIGURE 1:**
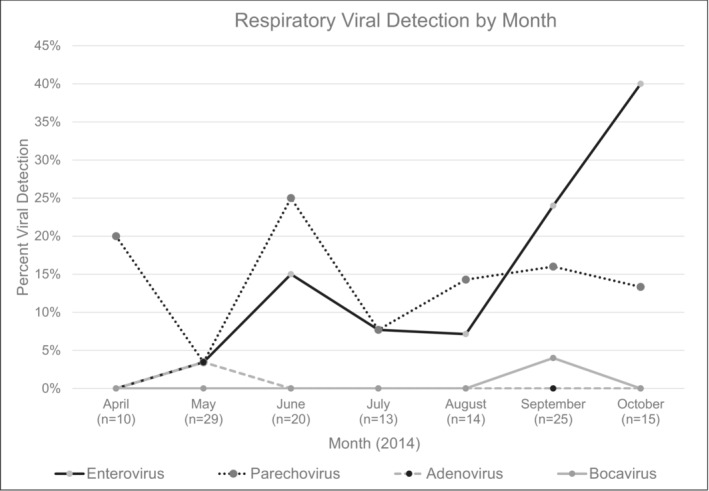
The percentage of plasma and/or cerebrospinal fluid samples positive for a respiratory virus by rtPCR by the month of sample collection. Four viruses are described (enterovirus, parechovirus, adenovirus, and bocavirus). Respiratory syncytial virus and rhinovirus were also tested for but are not described because their detection rates were 0% throughout the study period.

**FIGURE 2:**
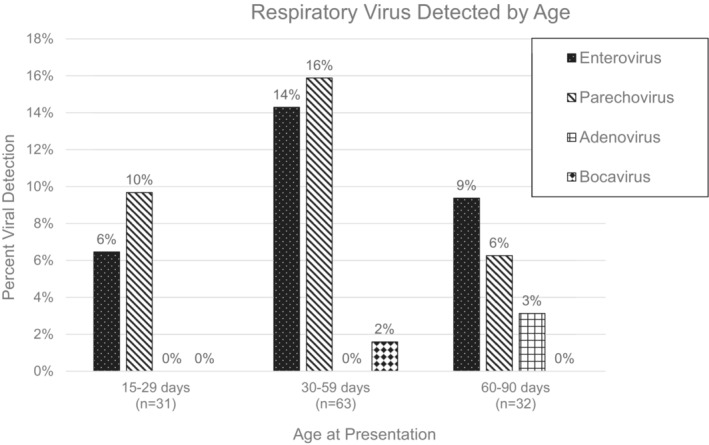
The percentage of plasma and/or cerebrospinal fluid samples positive for a respiratory virus by rtPCR according to the age group of the infants. Four viruses are described (enterovirus, parechovirus, adenovirus, and bocavirus). Respiratory syncytial virus and rhinovirus were also tested for but are not described because their detection rates were 0% throughout the study period.

Of the 126 infants, 30 (24%) had a positive viral rtPCR test from any sample type. Viral positivity was highest in plasma samples at 25% (27 of 107) compared with CSF samples at 15% (nine of 62). The most commonly identified viruses were parechovirus (12%, 15 infants) and enterovirus (11%, 14 infants). Bocavirus type I and adenovirus were each identified in the plasma sample of separate infants. No infants had plasma or CSF samples positive for RSV or rhinovirus (Table 2). Of the 43 infants with both plasma and CSF samples available, 15 infants (35%) had a virus detected in their plasma, while only eight infants (19%) had a virus detected in their CSF (Table 2). Six infants (14%) had a virus detected in both their plasma and CSF samples; the same virus was identified in both sample types for five of the six infants. The remaining infant had bocavirus detected in the plasma and parechovirus detected in the CSF. Enterovirus was most commonly detected in infants enrolled in September and October, while parechovirus was present throughout the recruitment period (Figure 1). Respiratory virus positivity was highest in infants 30–59 days (30%) and lowest in infants 15–29 days (16%) (Figure 2).

**TABLE 2:** Respiratory viral detection by specimen type.

		Positive testing in each sample type		Positive testing in infants with both plasma and CSF samples available	
Respiratory virus detected		107 infants had an available plasma sample	62 infants had an available CSF sample	43 infants had both plasma and CSF samples available	
				Plasma	CSF
RSV	*n* (%)	0 (0%)	0 (0%)	0 (0%)	0 (0%)
Rhinovirus	*n* (%)	0 (0%)	0 (0%)	0 (0%)	0 (0%)
Enterovirus	*n* (%)	12 (11%)	3 (5%)	8 (19%)	2 (5%)
Parechovirus	*n* (%)	13 (12%)	6 (10%)	6 (14%)	6 (14%)
Adenovirus	*n* (%)	1 (1%)	0 (0%)	0 (0%)	0 (0%)
Bocavirus	*n* (%)	1 (1%)	0 (0%)	1 (2%)	0 (0%)
**All viruses**	***n* (%)**	**27 (25%)**	**9 (15%)**	**15 (35%)** ^**a**^	**8 (19%)**

Abbreviations: CSF, cerebrospinal fluid; RSV, respiratory syncytial virus.

^a^ One infant had parechovirus detected in the CSF and bocavirus detected in the plasma. Additionally, for enterovirus only one infant of the 43 had enterovirus detected in both plasma and CSF samples, while for parechovirus five infants had parechovirus detected in both plasma and CSF samples.

### 3.3 SBIs

SBIs occurred in 21% (26) of infants in our cohort. bUTIs were most common, occurring in 18% (23) of infants, followed by bacteremia in 4% (5) of infants and bacterial meningitis in 1% (1) of infants (Table 3). See Table S1 for a detailed description of all cases of SBI. SBIs occurred in 3% (one of 30) of infants with a respiratory virus detected in either their plasma or CSF, compared to 26% (25 of 96) of infants with negative viral testing (*p* = 0.008). This difference was driven by infants with bUTI, as 24% of the negative respiratory viral testing group had a bUTI, while the positive respiratory viral testing group had 0% (Table 3). Of note, the infant with bacterial meningitis in the virus detected group was rehospitalized 8 days after discharge for the original febrile presentation and initially had a full sepsis (urine, blood, and CSF bacterial cultures) workup that was negative. It is difficult to determine whether this SBI was present in early stages at the initial evaluation; however, it did meet our initial criteria for readmission.

**TABLE 3:** Viral and bacterial coinfection.

		Virus detected (*n* = 30)	Virus not detected (*n* = 96)	*p*‐value
UTI	*n* (%)	0 (0%)	23 (24%)	0.004
Bacteremia	*n* (%)	0 (0%)	5 (5%)	NS
Bacterial meningitis	*n* (%)	1 (3%)^a^	0 (0%)	NS
All SBIs	*n* (%)	1 (3%)	25 (26%)	0.008

Abbreviations: NS, not significant; SBI, serious bacterial infection; UTI, urinary tract infection.

^a^ This patient returned 8 days after discharge with GBS meningitis.

## 4 DISCUSSION

Enterovirus and parechovirus were frequently detected in the plasma and CSF samples of our cohort. Parechovirus was the most commonly detected virus closely followed by enterovirus. These data add to the growing body of literature that suggest parechovirus is an important pathogen to be considered in the neonatal fever evaluation.^28^, ^29^ This was specifically illustrated in 2022 with the release of a health advisory by the Centers for Disease Control and Prevention (CDC) that detailed recent cases of human parechovirus in the United States.^30^ While detection of parechovirus has been increasing, our data illustrates that parechovirus has been affecting young infants for years. Parechovirus testing should be considered in situations where enterovirus testing is being performed. A recently published study from the Pediatric Emergency Care Applied Research Network (PECARN) detailed the viral testing of 4778 infants from 26 EDs across the United States.^4^ The most commonly tested viruses in the plasma and CSF of febrile infants ≤90 days were enterovirus and herpes simplex virus; however, testing for parechovirus was rarely, if at all, performed.^4^ This may be a missed opportunity to identify an infant's etiology of fever. Our data suggest that parechovirus is as common as enterovirus in the plasma and CSF samples of febrile infants ≤90 days, and it should be considered in our diagnostic repertoire.

Plasma sampling for enterovirus and parechovirus may provide more diagnostic value than CSF testing alone, especially in situations where CSF is not being obtained. Overall, in our cohort, 24% of febrile infants 15–90 days had a virus detected in either the plasma or the CSF. Additionally, 25% of plasma samples were positive, while only 15% of CSF samples were positive. In our cohort, only 7% of infants had a positive viral test in the CSF without viral detection in the plasma, which is similar to the 4% described in another recent study.^28^ Together, these data suggest that enterovirus and parechovirus detection in young febrile infants could be driven by plasma sampling alone rather than CSF sampling. One factor to note is that for this to be practical in the clinical setting, it would be reasonable to employ multiplex testing; however, this would need to be balanced with a potential reduction in sensitivity when multiple targets are employed in rtPCR.

Finally, similar to other cohorts, we found a decreased risk in the occurrence of UTI and overall SBI (though numbers are limited for invasive bacterial infections) in infants with a virus detected in their plasma or CSF. While this finding will not enable clinicians to discontinue antibiotics with the occurrence of a positive viral test, it does support the idea that in the context of viral testing in the blood and CSF, viral/bacterial co‐infections are relatively uncommon. This differs from respiratory viral testing, where half of all SBIs are associated with a positive viral test from respiratory secretions.^5^ This suggests that respiratory viral testing in plasma and CSF may be more specific for the risk reduction of an SBI. More data are needed to understand the relative risks associated with a positive viral test in any specimen type. However, it is our hope that one day, viral testing in the context of a diagnostic algorithm will improve the diagnosis and management of young febrile infants by reducing unnecessary antibiotic use and the need for invasive procedures.

## 5 LIMITATIONS

This study has several limitations. The first is that paired plasma and CSF samples were not available for each infant. Because lumbar punctures are invasive, they are not obtained in febrile infants ≤90 days when not clinically indicated. Additionally, because there are limitations on the amounts of plasma and CSF that can be drawn from young infants, there were some instances of insufficient sample volume for our examination, after the clinical studies were completed. Unavailable samples are unavoidable in this infant population; however, we did provide a description of viruses detected in those infants who had both plasma and CSF samples available, and they were representative of those viruses detected in febrile infants with only an available plasma or CSF sample. Additionally, were unable to correlate these results to viral testing in the respiratory tract because only 31/126 infants received a nasal swab/nasal wash for testing. Of these 31 infants, three were positive for parainfluenza by PCR, five were positive by viral culture (four picornavirus, one cytomegalovirus), and one was positive for RSV by rapid antigen testing. Because of the differing modalities of respiratory viral testing used in the clinical setting, it is difficult to draw conclusions on correlations between these sample types; however, one of the infants with a positive viral culture for picornavirus did have a positive PCR for enterovirus in the blood. All others had negative blood and CSF when available.

The second is that recruitment only occurred from April 2014 to October 2014. This period limited the range of viruses that were most likely to be detected. The recruitment period may have increased or decreased the incidence of particular viruses, as RSV generally circulates during the fall and winter months and enterovirus was generally detected in the summer and fall months, prior to the COVID‐19 pandemic.

Finally, the age range of these infants was 15 days through 90 days. Because the highest risk of SBI is in the first 4 weeks of life, the exclusion of infants ≤14 days could have reduced our detection of SBIs. However, the peak rates of SBIs are in the third week of life, which was included in our cohort, and our SBI detection of 21% is higher than typically described.^31^ This was likely due to the increased likelihood of blood and CSF sampling in infants with an SBI compared to those with normal screening results.

## 6 CONCLUSIONS

Our findings support the use of molecular diagnostics to include the identification of parechovirus in febrile infants ≤90 days. Additionally, these data support the utilization of blood specimen collection to diagnose parechovirus and enterovirus infection in febrile infants ≤90 days of life.

## AUTHOR CONTRIBUTIONS

**Erin G. Nicholson:** Conceptualization; data curation; investigation; methodology; project administration; writing—original draft. **Vasanthi Avadhanula:** Conceptualization; methodology; project administration; resources; supervision; writing—review and editing. **Leila Sahni:** Conceptualization; data curation; investigation; project administration; resources; writing—review and editing. **Laura L. Ferlic‐Stark:** Data curation; formal analysis; software; validation; writing—review and editing. **Lauren Maurer:** Investigation; methodology; project administration; writing—review and editing. **Julie Boom:** Conceptualization; investigation; methodology; project administration; resources; supervision; writing—review and editing. **Pedro A. Piedra:** Conceptualization; investigation; methodology; resources; supervision; writing—original draft.

## CONFLICT OF INTEREST STATEMENT

The authors do not have any conflicts of interest to disclose with regard to the work.

### PEER REVIEW

The peer review history for this article is available at https://www.webofscience.com/api/gateway/wos/peer-review/10.1111/irv.13250.

## ETHICS STATEMENT

The study was approved by the institutional review board (IRB) at Baylor College of Medicine. Each participant's legally authorized representative provided informed consent. No material included in this manuscript has been reproduced from other sources.

## Supporting information


**Table S1.** Detailed Description of all Instances of Recorded SBI

## Data Availability

The data that support the findings of this study are available on request from the corresponding author. The data are not publicly available due to privacy or ethical restrictions and release of data will be contingent upon approval from appropriate ethical review boards.

## References

[1] Dagan R , Powell KR , Hall CB , Menegus MA . Identification of infants unlikely to have serious bacterial infection although hospitalized for suspected sepsis. J Pediatr. 1985;107(6):855‐860. doi:10.1016/S0022-3476(85)80175-X 4067741

[2] Byington CL , Enriquez FR , Hoff C , et al. Serious bacterial infections in febrile infants 1 to 90 days old with and without viral infections. Pediatrics. 2004;113(6):1662‐1666. doi:10.1542/peds.113.6.1662 15173488

[3] Greenhow TL , Hung Y‐Y , Herz AM , Losada E , Pantell RH . The changing epidemiology of serious bacterial infections in young infants. Pediatr Infect Dis J. 2014;33(6):595‐599. doi:10.1097/INF.0000000000000225 24326416

[4] Mahajan P , Browne LR , Levine DA , et al. Risk of bacterial coinfections in febrile infants 60 days old and younger with documented viral infections. J Pediatr. 2018;203:86‐91.e2. doi:10.1016/j.jpeds.2018.07.073 30195552 PMC7094460

[5] Nicholson EG , Avadhanula V , Ferlic‐Stark L , Patel K , Gincoo KE , Piedra PA . The risk of serious bacterial infection in febrile infants 0–90 days of life with a respiratory viral infection. Pediatr Infect Dis J. 2019;38(4):355‐361. doi:10.1097/INF.0000000000002165 30882724

[6] Kidszun A , Klein L , Winter J , et al. Viral infections in neonates with suspected late‐onset bacterial sepsis—a prospective cohort study. Am J Perinatol. 2016;34(1):1‐7. doi:10.1055/s-0036-1584150 27182999 PMC7171717

[7] Doby EH , Stockmann C , Korgenski EK , Blaschke AJ , Byington CL . Cerebrospinal fluid pleocytosis in febrile infants 1–90 days with urinary tract infection. Pediatr Infect Dis J. 2013;32(9):1024‐1026. doi:10.1097/INF.0b013e31829063cd 23584580 PMC3755104

[8] Al‐Mously N , Azizalrahman A , Al HTM , Altamimi SA . Molecular epidemiology of respiratory viruses in febrile infants under 90 days attending pediatric emergency department. Am J Infect Dis Microbiol. 2016;4(2):35‐40. doi:10.12691/AJIDM-4-2-3

[9] Titus MO , Wright SW . Prevalence of serious bacterial infections in febrile infants with respiratory syncytial virus infection. Pediatrics. 2003;112(2):282‐284. doi:10.1542/peds.112.2.282 12897274

[10] American College of Emergency Physicians Clinical Policies Committee , American College of Emergency Physicians Clinical Policies Subcommittee on Pediatric Fever . Clinical policy for children younger than three years presenting to the emergency department with fever. Ann Emerg Med. 2003;42(4):530‐545. doi:10.1067/S0196-0644(03)00628-0 14520324

[11] Blaschke AJ , Korgenski EK , Wilkes J , et al. Rhinovirus in febrile infants and risk of bacterial infection. Pediatrics. 2018;141(2):e20172384. doi:10.1542/peds.2017-2384 29343585 PMC5810600

[12] Burstein B , Dubrovsky AS , Greene AW , Quach C . National Survey on the impact of viral testing for the ED and inpatient management of febrile young infants. Hosp Pediatr. 2016;6(4):226‐233. doi:10.1542/hpeds.2015-0195 27005580

[13] Byington CL , Taggart EW , Carroll KC , Hillyard DR . A polymerase chain reaction‐based epidemiologic investigation of the incidence of nonpolio enteroviral infections in febrile and afebrile infants 90 days and younger. Pediatrics. 1999;103(3):E27. doi:10.1542/peds.103.3.e27 10049983

[14] Byington CL , Reynolds CC , Korgenski K , et al. Costs and infant outcomes after implementation of a care process model for febrile infants. Pediatrics. 2012;130(1):e16‐e24. doi:10.1542/peds.2012-0127 22732178 PMC4074609

[15] Pantell RH , Roberts KB , Adams WG , et al. Evaluation and management of well‐appearing febrile infants 8 to 60 days old. Pediatrics. 2021;148(2). doi:10.1542/PEDS.2021-052228 34281996

[16] Nigrovic LE , Kuppermann N , Neuman MI . Risk factors for traumatic or unsuccessful lumbar punctures in children. Ann Emerg Med. 2007;49(6):762‐771. doi:10.1016/J.ANNEMERGMED.2006.10.018 17321005

[17] Lafolie J , Labbé A , L'Honneur AS , et al. Assessment of blood enterovirus PCR testing in paediatric populations with fever without source, sepsis‐like disease, or suspected meningitis: a prospective, multicentre, observational cohort study. Lancet Infect Dis. 2018;18(12):1385‐1396. doi:10.1016/S1473-3099(18)30479-1 30389482 PMC7164799

[18] Sahni LC , Fischer RSB , Gorchakov R , et al. Arboviral surveillance among pediatric patients with acute febrile illness in Houston, Texas. Am J Trop Med Hyg. 2018;99(2):413‐416. doi:10.4269/ajtmh.17-0891 29869599 PMC6090340

[19] Bennett BL , Garofalo RP , Cron SG , et al. Immunopathogenesis of respiratory syncytial virus bronchiolitis. J Infect Dis. 2007;195(10):1532‐1540. doi:10.1086/515575 17436234

[20] Mansbach JM , Piedra PA , Stevenson MD , et al. Prospective multicenter study of children with bronchiolitis requiring mechanical ventilation. Pediatrics. 2012;130(3):e492‐e500. doi:10.1542/peds.2012-0444 22869823 PMC3428760

[21] Selvaraju S , Nix A , Oberste S , Selvarangan R . Optimization of a combined human parechovirus‐enterovirus real‐time reverse transcription‐PCR assay and evaluation of a new parechovirus 3‐specific assay for cerebrospinal fluid specimen testing. J Clin Microbiol. 2013;51(2):452‐458. doi:10.1128/JCM.01982-12 23175256 PMC3553926

[22] Harris PA , Taylor R , Minor BL , et al. The REDCap consortium: building an international community of software platform partners. J Biomed Inform. 2019;95:103208. doi:10.1016/j.jbi.2019.103208 31078660 PMC7254481

[23] Harris PA , Taylor R , Thielke R , Payne J , Gonzalez N , Conde JG . Research electronic data capture (REDCap)‐‐a metadata‐driven methodology and workflow process for providing translational research informatics support. J Biomed Inform. 2009;42(2):377‐381. doi:10.1016/j.jbi.2008.08.010 18929686 PMC2700030

[24] Krief WI , Levine DA , Platt SL , et al. Influenza virus infection and the risk of serious bacterial infections in young febrile infants. Pediatrics. 2009;124(1):30‐39. doi:10.1542/peds.2008-2915 19564280

[25] Levine DA , Platt SL , Dayan PS , et al. Risk of serious bacterial infection in young febrile infants with respiratory syncytial virus infections. Pediatrics. 2004;113(6):1728‐1734. doi:10.1542/peds.113.6.1728 15173498

[26] R Core Team . R: a language and environment for statistical computing. R Foundation for Statistical Computing. https://www.r-project.org/. Published 2017.

[27] StataCorp . Stata statistical software: release 14. College Station, TX: StataCorp LP. 2015. 2015. 10.2307/2234838

[28] Tomatis Souverbielle C , Wang H , Feister J , et al. Year‐round, routine testing of multiple body site specimens for human parechovirus in young febrile infants. J Pediatr. 2021;229:216‐222.e2. doi:10.1016/J.JPEDS.2020.10.004 33045237 PMC7546655

[29] Britton PN , Jones CA , Macartney K , Cheng AC . Parechovirus: an important emerging infection in young infants. Med J Aust. 2018;208(8):365‐369. doi:10.5694/MJA18.00149 29716506

[30] Recent reports of human parechovirus (PeV) in the United States—2022. HAN Archive ‐ 00469|Health Alert Network (HAN). https://emergency.cdc.gov/han/2022/han00469.asp. Published 2022. Accessed January 31, 2023.

[31] Aronson PL , Thurm C , Alpern ER , et al. Variation in care of the febrile young infant <90 days in US pediatric emergency departments. Pediatrics. 2014;134(4):667‐677. doi:10.1542/peds.2014-1382 25266437

