# Supplementary material for: Respiratory viral detection in the plasma and cerebrospinal fluid (CSF) of young febrile infants
Source: Influenza Other Respir Viruses. 2024 Feb 1;18(2):e13250. doi: 10.1111/irv.13250 (PMC10831571; doi:10.1111/irv.13250)
Supplement: Supplementary file 1 — Table S1. Detailed Description of all Instances of Recorded SBI [file IRV-18-e13250-s001.docx]

| **Supplemental Table 1: Detailed Description of all Instances of Recorded SBI** | | | | | | | | | | | |
| --- | --- | --- | --- | --- | --- | --- | --- | --- | --- | --- | --- |
| **Age at Enrollment** | **Urine Nitrites** | **Urine Leukocyte esterase** | **Urine WBC** | **Urine Bacteria** | **Urine Culture Results** | **Blood culture Results** | **CSF Culture Results** | **SBI Type** | **Hospital PCR results** | **Laboratory Blood PCR Result** | **Laboratory CSF PCR Result** |
| 6 | Negative | Negative | Not Performed | Not Performed | >100,000 *Enterococcus* species | Coagulase Negative *Staphylococcus* | Negative | UTI | Enterovirus CSF PCR Negative | Negative | Negative |
| 19 | Positive | Positive | TNTC | Positive | >100,000 *E.coli* | Negative | Negative | UTI | Enterovirus and HSV CSF PCR Negative | Not Available | Negative |
| 20 | Positive | Positive | TNTC | Positive | >100,000 *E.coli* | Negative | Negative | UTI | Not Performed | Not Available | Negative |
| 21 | Negative | Positive | 5-10 | Positive | >100,000 *E.coli* | Negative | Negative | UTI | Not Performed | Negative | Negative |
| 29 | Negative | Positive | 21-50 | Positive | 10-50,000 *E.coli* | Alpha *Streptococcus* | Negative | UTI/  Bacteremia | Enterovirus and HSV CSF PCR Negative | Negative | Negative |
| 30 | Positive | Positive | 21-50 | Positive | >100,000 *E.coli* | Negative | Negative | UTI | Not Performed | Not Available | Negative |
| 31 | Positive | Positive | TNTC | Positive | 50-100,000 *E.coli* | E.coli | Negative | UTI/  Bacteremia | Not Performed | Negative | Negative |
| 32 | Negative | Positive | TNTC | Positive | >100,000 *E.coli* | Negative | Negative | UTI | Not Performed | Negative | Negative |
| 33 | Negative | Negative | 0-4 | Positive | <10,000 Regional Bacteria | Negative | Negative | Bacterial Meningitis ❖ | Not Performed | Negative | CSF Parechovirus Positive |
| 36 | Negative | Negative | Not Performed | Not Performed | Negative | Coagulase Negative *Staphylococcus* | Not Performed | Bacteremia ✼ | Not Performed | Negative | Not Available |
| 37 | Negative | Negative | 0-4 | Positive | Negative | Enterobacter species | Negative | Bacteremia | Enterovirus CSF PCR Negative | Not Available | Negative |
| 38 | Negative | Positive | 51-100 | Positive | 50-100,000 *E.coli* | Coagulase Negative *Staphylococcus* | Negative | UTI | Not Performed | Negative | Negative |
| 43 | Negative | Positive | TNTC | Positive | 50-100,000 *E.coli* | Negative | Negative | UTI | Not Performed | Not Available | Negative |
| 44 | Negative | Negative | Not Performed | Not Performed | 50-100,000 *Proteus* Species | *Bacillus* non-anthracis | Negative | UTI | Not Performed | Negative | Not Available |
| 47 | Positive | Positive | 51-100 | Positive | >100,000 *E.coli* | *E.coli* | Negative | UTI/  Bacteremia | HSV CSF PCR Negative | Negative | Negative |
| 50 | Negative | Positive | Not Performed | Not Performed | 10-50,000 *E.coli* | Negative | Negative | UTI | Enterovirus and HSV CSF PCR Negative | Negative | Negative |
| 53 | Positive | Positive | 11-20 | Positive | 50-100,000 *E.coli* | Negative | Negative | UTI | Not Performed | Negative | Negative |
| 56 | Positive | Positive | 51-100 | Positive | >100,000 *E.coli* | Negative | Not Performed | UTI | Not Performed | Negative | Not Available |
| 58 | Negative | Positive | TNTC | Positive | 50-100,000 *E.coli* | Negative | Negative | UTI | Not Performed | Negative | Negative |
| 64 | Negative | Positive | 21-50 | Positive | 10-50,000 *E.coli* | Coagulase Negative *Staphylococcus* | Not Performed | UTI | Not Performed | Negative | Not Available |
| 70 | Positive | Positive | TNTC | Positive | >100,000 *E.coli* | Negative | Negative | UTI | Not Performed | Not Available | Negative |
| 71 | Negative | Positive | 21-50 | Positive | >100,000 *E.coli* | Negative | Not Performed | UTI | Not Performed | Negative | Not Available |
| 72 | Negative | Positive | TNTC | Positive | >100,000 *E.coli* | Negative | Not Performed | UTI | Not Performed | Negative | Not Available |
| 79 | Negative | Positive | TNTC | Not Performed | 10-50,000 *E.coli* | Negative | Not Performed | UTI | Not Performed | Negative | Not Available |
| 81 | Negative | Positive | 51-100 | Not Performed | >100,000 *E.coli* | Negative | Not Performed | UTI | Not Performed | Negative | Not Available |
| 87 | Negative | Positive | TNTC | Positive | 50-100,000 *E.coli* | Negative | Not Performed | UTI | Respiratory Viral Panel Negative | Negative | Not Available |
| ❖ This patient returned 8 days after discharge with GBS meningitis | | | | | | | | | | | |
| ✼ This patient had multiple positive central line cultures for coagulase negative *staphylococcus* | | | | | | | | | | | |
| Abbreviations: Serious Bacterial Infection (SBI), White Blood Cells (WBC), Cerebrospinal Fluid (CSF), Polymerase Chain Reaction (PCR), Too Numerous to Count (TNTC), Urinary Tract Infection (UTI), Herpes Simplex Virus (HSV) | | | | | | | | | | | |
|  |  |  |  |  |  |  |  |  |  |  |  |
